# Integrated Transcriptomic and Proteomic Analyses of Low-Nitrogen-Stress Tolerance and Function Analysis of ZmGST42 Gene in Maize

**DOI:** 10.3390/antiox12101831

**Published:** 2023-10-05

**Authors:** Jiao Li, Tinashe Zenda, Songtao Liu, Anyi Dong, Yafei Wang, Xinyue Liu, Nan Wang, Huijun Duan

**Affiliations:** 1 State Key Laboratory of North China Crop Improvement and Regulation, Hebei Agricultural University, Baoding 071001, China; lj337871790219@163.com (J.L.); zenda@hebau.edu.cn (T.Z.); 18331220513@163.com (A.D.); wyf360536991@163.com (Y.W.); lxy1696164468@163.com (X.L.); 2 North China Key Laboratory for Crop Germplasm Resources of the Education Ministry, Hebei Agricultural University, Baoding 071001, China; 3 Department of Crop Genetics and Breeding, College of Agronomy, Hebei Agricultural University, Baoding 071001, China; 4 Hebei Key Laboratory of Quality & Safety Analysis-Testing for Agro-Products and Food, Academy of Agriculture and Forestry Sciences, Hebei North University, Zhangjiakou 075000, China; m15028293845@163.com

**Keywords:** low-nitrogen stress, grain filling stage, proteome, transcriptome, glutathione transferase 42 (GST42), maize

## Abstract

Maize (*Zea mays* L.) is one of the major staple crops providing human food, animal feed, and raw material support for biofuel production. For its growth and development, maize requires essential macronutrients. In particular, nitrogen (N) plays an important role in determining the final yield and quality of a maize crop. However, the excessive application of N fertilizer is causing serious pollution of land area and water bodies. Therefore, cultivating high-yield and low-N-tolerant maize varieties is crucial for minimizing the nitrate pollution of land and water bodies. Here, based on the analysis of the maize leaf transcriptome and proteome at the grain filling stage, we identified 3957 differentially expressed genes (DEGs) and 329 differentially abundant proteins (DAPs) from the two maize hybrids contrasting in N stress tolerance (low-N-tolerant XY335 and low-N-sensitive HN138) and screened four sets of low-N-responsive genes and proteins through Venn diagram analysis. We identified 761 DEGs (253 up- and 508 down-regulated) specific to XY335, whereas 259 DEGs (198 up- and 61 down-regulated) were specific to HN138, and 59 DEGs (41 up- and 18 down-regulated) were shared between the two cultivars under low-N-stress conditions. Meanwhile, among the low-N-responsive DAPs, thirty were unique to XY335, thirty were specific to HN138, and three DAPs were shared between the two cultivars under low-N treatment. Key among those genes/proteins were leucine-rich repeat protein, DEAD-box ATP-dependent RNA helicase family proteins, copper transport protein, and photosynthesis-related proteins. These genes/proteins were involved in the MAPK signaling pathway, regulating membrane lipid peroxidation, and photosynthesis. Our results may suggest that XY335 better tolerates low-N stress than HN138, possibly through robust low-N-stress sensing and signaling, amplified protein phosphorylation and stress response, and increased photosynthesis efficiency, as well as the down-regulation of ‘lavish’ or redundant proteins to minimize N demand. Additionally, we screened glutathione transferase 42 (*ZmGST42*) and performed physiological and biochemical characterizations of the wild-type (B73) and *gst42* mutant at the seedling stage. Resultantly, the wild-type exhibited stronger tolerance to low N than the mutant line. Our findings provide a better understanding of the molecular mechanisms underlying low-N tolerance during the maize grain filling stage and reveal key candidate genes for low-N-tolerance breeding in maize.

## 1. Introduction

Nitrogen (N) is a major limiting nutrient for plant growth and development, especially for major food crops such as maize (*Zea mays* L.) [1]. It is one of the essential macronutrients for plant growth and development whose availability determines plant productivity [2,3]. Thus, N availability can influence maize plant growth and productivity [4]. In order to meet the required crop production levels and fulfill the global food demands, several million tons of N fertilizer are applied to croplands annually, raising the global demand for N fertilizers [3]. However, the excessive, and inappropriate, application of N fertilizer leads to low N use efficiency (NUE), where 50–70% of the applied N is lost to the surrounding environment, resulting in serious environmental problems including soil acidification and the eutrophication of water bodies [3,5]. Excess N and its related negative impacts on the environment have become a global concern. Hence, new and efficient strategies to improve NUE and increase cultivars’ tolerance to low N levels have been proposed in order to improve crop yield under limited N levels [1].

Maize is one of the world’s major food crops, serving as human food, animal feed, and raw material for biofuel production [6]. Maize production largely relies on N nutrient fertilizer additions for higher yields. Thus, a large amount of N fertilizer has been used in maize production [4]. However, despite the positive gains in maize yield increase, excessive addition of N has exacerbated N nutrient loss via leaching, leading to the environmental problems identified above [1,7]. Plausibly, therefore, reducing the input of N fertilizer and cultivating low-N-tolerant or N-efficient varieties are the new strategies to increase maize yield with minimal N fertilizer additions while reducing environmental pollution and cutting production costs [8,9]. 

The maize grain filling stage is the key period when the biomass production and NUE values of different genotypes are different [10]. During this stage, there are differences in maize use of carbon (C) and N, whereby varieties with high NUE have strong photosynthetic C and N assimilation, as well as C and N metabolism capacity, which can meet the requirements of photosynthetic C and N assimilation [11]. Therefore, the lack of N during the grain filling stage will have a great impact on the final weights of maize kernels.

At present, there have been many studies on the analysis of maize response to low-N stress, but most of these studies have mainly focused on physiological and biochemical analyses [12,13,14]. For example, an experiment using maize genotype Denghai 618 with and without N fertilizer in the field showed that the photosynthetic parameters were significantly reduced under N deficiency, which was related to leaf senescence [13]. Under low-N treatment, the application of melatonin improved the growth of plant seedlings and activated superoxide dismutase (SOD), peroxidase (POD), and catalase (CAT) [14]. The maize leaf transcriptomes of N-sensitive inbred line B73 and tolerant inbred line Mo17 under sufficient-N and low-N conditions at the seedling stage indicated that DEGs affected by N availability and genotype were mainly concentrated in amino acid metabolism and photosynthesis, secondary metabolism, and gene replication and expression pathways [12]. However, there has been relatively few integrated transcriptomics and proteomics studies revealing the pathways related to low-N stress in maize. Therefore, it is important to integrate these approaches to study the molecular mechanisms underpinning summer maize tolerance to low-N stress. 

Previously, we conducted a comparative analysis of the yield and physiological and molecular responses of two contrasting maize genotypes (tolerant XY335 and susceptible HN138) to low-N stress, and we observed that XY335 had higher grain yield, greater shoot N uptake, and NUE than HN138 [15]. Additionally, under low-N stress, the activity of nitrate reductase (NR) and glutathione S-transferase (GST) were enhanced in XY335, which had a positive effect on the grain yield. However, the role and expression patterns of NR and GST genes in maize tolerance to low N are not yet clear. 

GST, a superfamily of enzymes, mainly plays key roles in detoxification and defense against harmful substances [16]. GST catalyzes the covalent binding of reduced glutathione (GSH) and hydrophobic and electrophilic substrates to form conjugates, which are isolated in vacuoles or transferred to apoplasts, thereby degrading endogenous and foreign harmful substances [17]. In plants, the expression of GST mainly occurs during plant development (cell division and senescence), changes in the external environment, and the spraying of chemical substances [18,19]. At present, GST has been found to be induced by a variety of external stress factors such as oxidative stress, drought stress, heavy metals, etc. [20]. However, the expression behavior and gene function of *ZmGST* in maize drought stress tolerance are still not yet clear. Therefore, understanding the mechanism and gene expression pattern of the *ZmGST* gene in response to drought stress is important for improving low-N-stress tolerance

Meanwhile, RNA sequencing (RNA-seq) is a high-throughput sequencing technology with high accuracy and low cost and has been widely used in gene expression analysis in plants [21]. Additionally, since proteins are directly involved in the stress responses of plants, the study of proteomics is helpful to analyze the relationship between protein changes and plant stress tolerance [22,23]. Thus, proteomics analysis of the molecular mechanisms of maize tolerance to low-N stress has become important [24]. Through the integrated analysis of the transcriptome and proteome changes in response to stress at different levels, gene expression and protein accumulation/abundances can reveal new information or provide more complete details about how plants tolerate respective stress.

Here, we studied the leaf transcriptome and protein expression profiles of Xianyu 335 (XY335) and Huanong 138 (HN138) maize cultivars in response to low-N stress during the grain filling stage. Our main objectives of this study were: (1) to screen the key N-metabolism-related genes and proteins responsive to low N availability through the comparative analysis of two contrasting maize hybrids; (2) to reveal the molecular mechanisms underlying the response of a low-N-tolerant maize hybrid to low-N stress; and (3) to analyze the function of the *ZmGST42* gene in low-N-stress response in maize. Our results improve our understanding of the molecular mechanisms underlying low-N tolerance at the grain filling stage and provide candidate genes for low-N-tolerance breeding in maize.

## 2. Materials and Methods

### 2.1. Plant Materials and Treatment

Two maize hybrid cultivars contrasting in low-N tolerance (low-N-tolerant XY335 and low-N-sensitive HN138) were used in this study [15]. The experiment was carried out at Xinji Experimental Station (43°31′ N, 124°48′ E) at Hebei Agricultural University during 2018 cropping season. The top 0–20 cm of the soil used contained 17.79 g·kg^−1^ organic matter, 1.21 g·kg^−1^ total N, 64.9 mg.kg^−1^ alkali hydrolyzed N, 23.8 mg·kg^−1^ available phosphorus, and 120.6 mg·kg^−1^ available potassium. The experiment adopted a split plot design, with varieties as the main plot and N fertilizer as the sub-plot. There were 2 hybrids for testing—XY335 and HN138—and two levels of N supply: N0 (0 kg N ha^−1^) and N240 (240 kg N ha^−1^), replicated three times. Each plot had 6 rows, with the row length measuring 20 m, and the row spacing being 60 cm, giving the plot area of 72 m^2^. The planting density was 67,500 plants ha^−1^. All plots were supplemented with basic phosphorus (P_2_O_5_ 90 kg ha^−1^) and potassium (K_2_O 120 kg ha^−1^). We used the recommended conventional methods for other cultivation practices, such as irrigation (pre-planting and heading period), weed removal, and chemical control of pests and diseases. Nitrogen fertilizer used was urea (46% N), and 50% portions were applied before sowing and at the V12 period, respectively. At the R3 stage (21 days after silking) [25], the leaf tissues (of ~10 cm, from the middle of the ear) were collected from the control and treatment conditions, at three biological replicates, with each replicate constituted by at least five plants. The samples were immediately frozen in liquid N for subsequent proteomics analysis.

### 2.2. Transcriptomic Analysis of the Maize Leaf Samples 

Four treatment combinations, viz., XY335 under sufficient N (control) (XYC), XY335 under low-N treatment (XYT), HN138 under sufficient N (control) (HNC), and HN138 under low-N treatment (HNT), were set [24]. Each treatment was replicated thrice to give 12 samples for RNA sequencing. The uppermost fully expanded leaves were selected for sample collection, with at least five leaves from different plants constituting a sample. Library sequencing was handled by Shanghai Meiji Biology Company (Shanghai, China). Comparisons between these treatments were made to obtain different treatment comparisons/groups as follows: XYT_XYC, HNT_HNC, HNC_XYC, and XYT_HNT. Sequencing libraries for the differentially expressed genes (DEGs) were constructed as per the expected standards of RNA-seq transcriptome libraries. Fragments per kilobases of exon model per million mapped reads (FPKM) method [26] was used to measure transcript abundances, and FPKM values ≥ 1 were used to determine genes expressed (for sequence reads mapping details, see descriptions in our previous paper [27]). Gene differential analysis of two experimental/treatment groups was performed using DESeq R [28]. Student’s *t*-test was used to calculate the *p*-values (threshold ≤ 0.05), which were then adjusted for false discovery rate (FDR) [29] to FDR < 0.001 |log2 FC| [27].

### 2.3. Proteomic Analysis of the Maize Leaf Samples

Similar to transcriptomics, four treatment combinations were set up, viz., XY335 under sufficient N (control) (XYC), XY335 under low-N treatment (XYT), HN138 under sufficient N (control) (HNC), and HN138 under low-N treatment (HNT) [24]. Each of these treatments were replicated three times, giving a total of 12 samples that were used for proteomic sequencing. The uppermost fully expanded leaves were selected for sample collection, with five plants constituting a replicate. Sequencing was conducted by Shanghai Meiji Biology Company (Shanghai, China). The total protein from all samples was extracted and digested into peptides, and the peptides were labeled with TMT reagent. Then, the samples were pooled together and analyzed using mass spectrometer. By means of Protein DiscovererTM Software (version 2.2), spectra were searched against the maize database (131585s), and the peptide spectral matches were authenticated based on q-values at 0.01 FDR. Proteins possessing at least one unique peptide were used for subsequent analysis. Student’s *t*-test was used to analyze the differentially abundant proteins (DAPs), with the proteins with a fold-change > 1.2 (up) or <0.83 (down) (*p*-value < 0.05) considered statistically significant [24]. Meanwhile, gene ontology (http://www.geneontology.org/, accessed on 16 May 2020) analysis was used for functional annotation and classification of DAPs. Further, Kyoto Encyclopedia of Genes and Genomes (KEGG, http://www.genome.jp/kegg/, accessed on 20 May 2020) database was used to assign DAPs to corresponding metabolic pathways, and hypergeometric test was used to analyze GO and KEGG enrichment of DAPs.

### 2.4. Correlation Analysis

Correlation analysis was performed on all data correlated at the protein and transcript levels. Based on the identification results, the qualified proteins and genes were screened out. The screening conditions were as follows: where the fold change in protein expression was ≥ 1.5 and *p* value ≤ 0.05; and where the fold change in gene expression was ≥ 2 and *p* value ≤ 0.001. The purpose was to identify differentially expressed genes and proteins for association analysis.

### 2.5. RNA Extraction, cDNA Synthesis, and qRT-PCR Verification of RNA-Seq Results

In order to verify the accuracy of the RNA-seq results, we randomly selected 12 genes for real-time quantitative PCR (qRT-PCR) detection. Primer Premier 5 Designer (Premier Biosoft International, Palo Alto, CA, USA) was used to design gene-specific primers for qRT-PCR analysis. The primer sequences of the 12 genes are shown in Supplementary Table S1. *GAPDH*, which has a stable expression in maize, was used as the reference gene. Total RNA was isolated from the non-stressed and stressed leaves of two hybrids (XY335 and HN138) at the grain filling stage (R3). RNA was then reverse transcribed into cDNA using the HiFiscript cDNA Synthesis Kit (CoWin Biosciences, Cambridge, MA, USA). The 20 µL total reaction volume contained 1 µL of template cDNA, 1 µL (100 µmol) of the front and back primers, and 10 µL of 2× M5 HiPer SYBR Premix EsTaq (with Tli RNaseH). Finally, a C1000 (CFX96 real-time system) thermal cycler (Bio-Rad, Shanghai, China) was used to perform qRT-PCR using 2× Fast Super EvaGreen^®^ qPCR Mastermix (US Everbright Inc., Suzhou, China). Each sample had three technical replicates.

### 2.6. Gene Function Analysis of ZmGST42 

#### 2.6.1. Materials and Methods

Combining the key genes identified in published literature and bioinformatics analysis, we selected *ZmGST42* (*glutathione transferase 42*) for gene function analysis. The ethyl methanesulfonate (EMS) mutagenized maize *gst42* mutant was purchased through the MEMD (Maize EMS-induced Mutant Database, http://elabcaas.cn/memd/) website [30]. It has B73 inbred line background. A 2 × 2 factorial design was used to establish the hydroponic experiment at the maize seedling stage, with two factors completely randomized. Factor A comprised two maize genotypes; factor B comprised two N treatments—low-N treatment (LN) at 0.04 mmol/L, and normal-N treatment (NN) at 4 mmol/L—replicated 3 times. Maize seeds with the same appearance and no mold and rot were selected, disinfected with 75% alcohol, washed with distilled water, placed in a germination tray covered with moist gauze, and germinated at 28 °C. When the root systems had grown to about 1 cm, seedlings with consistent growth and non-damaged root system were selected and carefully transferred to quartz sand to continue cultivation. When the seedlings had grown to third leaf stage, each seedling was transferred into a small hole (grid) with a diameter of 1.8 cm on a plastic gridded tray and fixed with a sponge. The experiment was set up with three replications, with each experimental unit comprising 12 plants grown in a plastic gridded tray (36 cm long, 24 cm wide and 10 cm high). The plastic gridded tray with a capacity of 5 L was supplied with Hoagland nutrient solution, and all seedlings of each treatment in a replicate were placed in that plastic box. When the seedlings had grown 4 leaves, they were treated with low-N stress. Plants were cultured in a culture room (day and night temperature of 26 °C and a light/dark cycle of 16/8 h). Ventilation was provided using an air pump for 5 h a day, and the nutrient solution was replaced every 2 days. After 12 days of stress treatment, various indices were measured.

The field experimental design and sampling of *ZmGST42* at the grain filling period were the same as above. After sampling, the samples were quick-frozen in liquid N and stored at −80 °C for subsequent experiments for the determination of physiological indices.

#### 2.6.2. Phenotypic Characterization of the WT Plant and Maize Mutant under Low-N Stress

Measurement of morphological traits started one day after LN treatment at the seedling stage and one day after silking at the grain filling stage. The plant height, root length, stem thickness, number of visible leaves, and the length and width of each leaf were measured every 3 days. Samples were taken on the 12th day of the stress treatment at the seedling stage and on the 21st day after silking at the grain filling stage. Three biological replicates (five plants constituted a replicate) were set up for each treatment to determine the above-ground traits and underground root traits.

For the measurement of plant height, we used a ruler to measure the distance between the highest point of the straightened leaf at the top and the ground. For stem thickness, we used a digital vernier caliper to measure the diameter of the lowest part of the stem. For green leaf area measurement, we used the following formula: green leaf area = leaf length × width × 0.75, wherein the width of the leaf is taken as the widest part of the leaf [31]. For determination of root traits, we used WinRHIZO software (Pro 2014b, Shanghai, China) to determine the total root length, projected area, and total root length from the image obtained from the Epson V700 (Seiko Epson, Suwa, Japan) scanner. We also measured the surface area, average diameter, and root volume.

For the determination of the above-ground parts, we used an electronic balance to measure the fresh weight of each above-ground part, then put it in an oven at 105 °C for 30 min and dried it to a constant weight at 80 °C before weighing again. For the roots, we used an electronic balance to measure each underground part’s fresh weight, then put it in an oven at 105 °C for 30 min, dried it to constant weight at 80 °C, and weighed again. Total dry matter was measured as the sum of root dry matter and above-ground dry matter. Root–shoot ratio was measured as follows: root dry matter as a ratio of the above-ground dry matter [32].

We measured SPAD values (to ascertain relative chlorophyll content) using a portable chlorophyll meter (SPAD-502Plus, Konica Minolta, Tokyo, Japan) [33]. Maize seedlings were subjected to low-N treatment for 12 days, and then, SPAD values were measured at the second top-most leaf, with 3 leaves per treatment replicate, and replicates were repeated thrice.

#### 2.6.3. Physiological and Biochemical Indices Determination

When the low-N stress-treatment was over and the measurement of each growth index was completed, the leaf samples of each treatment (the fully expanded fourth top-most leaf) were collected and 3 biological replicates constituted (with five plants per replicate). These samples were wrapped in tin foil and quickly frozen in liquid N and then stored at −80 °C for subsequent physiological and biochemical measurements.

The guaiacol method was used to determine the guaiacol peroxidase (POD) enzyme activity in maize leaves [34]. The nitroblue tetrazolium (NBT) photoreduction method was used to determine the SOD enzyme activity [35] and the thiobarbituric acid (TBA) method used to determine the MDA content [36]. Catalase (CAT) enzyme activity was measured through H_2_O_2_ decomposition [37] whereas nitrate reductase (NR) activity was measured according to Shaner and Boyer [38]. GS activity was measured using high performance liquid chromatography [25]. The activity levels of GST, GSH, γ-GT, and GOGAT were determined using the kit provided by the Suzhou Keming Biotechnology Co., Ltd. (Suzhou, China).

## 3. Results

### 3.1. RNA-Seq Transcriptome Analysis of the Two Maize Cultivars’ Response to Low N Treatment

**Figure 1:**
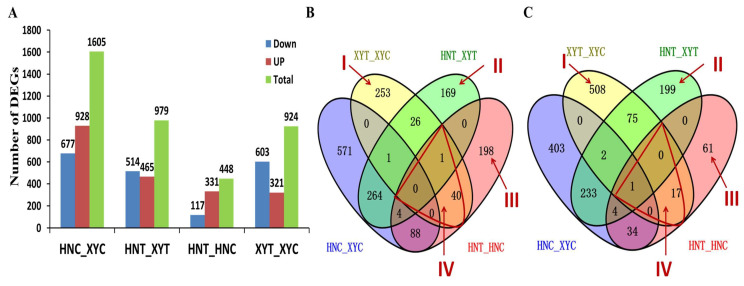
Analysis of DEGs identified in the four treatment comparisons. (**A**) Total number of DEGs identified in each comparison group based on expression type. Up-regulation means increased expression of the DEG, whereas down-regulation means reduced expression of the DEG; color representation: red—up-regulated and blue—down-regulated. (**B**) Venn diagram analysis of up-regulated DEGs. (**C**) Venn diagram analysis of down-regulated DEGs. The overlapping area of the Venn diagram indicates that there is an overlap in DEGs between the corresponding groups. Region I shows DEGs specifically expressed in XYT_XYC in response to low-N stress, region II contains DEGs uniquely expressed in HNT_HNC under low-N stress, and region IV shows DEGs overlapping between two hybrid lines under low-N stress.

The FPKM value was calculated by using the expression quantification software RSEM and the FPKM value was ≥1 as the standard. The treatment groups were compared and the numbers of DEGs in the groups are shown in Venn diagrams (Figure 1). The four treatments described in Section 2.2 above, viz., XYC, HNC, XYT, and HNT, were compared to form different comparisons (HNT_HNC, XYT_XYC, XYT_HNT, and HNC_XYC), and DEGs from these comparisons were analyzed. A total of 3956 DEGs were obtained from the four experimental comparisons (Figure 1A). Experimental comparison HNT_HNC had 448 DEGs, of which 331 genes were up-regulated and 117 down-regulated. Additionally, 924 DEGs were identified in the XYT_XYC comparison, of which 321 were up-regulated and 603 were down-regulated. There were 1605 DEGs in the comparison of the two hybrids under sufficient N (control) conditions (HNC_XYC), of which 928 were up-regulated and 677 were down-regulated. Meanwhile, the comparison of the two cultivars after low-N treatment (XYT_HNT) yielded 979 DEGs, of which 465 were up-regulated and 514 were down-regulated. These results showed that after low-N treatment, the number of down-regulated genes in XY335 was much higher than that in HN138 (Figure 1A). 

The Venn diagrams presented as Figure 1B,C show the comparative analysis of the up-regulated and down-regulated DEGs displayed in Figure 1A, with the important set of low-N-responsive DEGs marked as regions I-IV. The analysis showed that 761 DEGs were specifically expressed in XY335 under low-N conditions (region I), with 253 being up-regulated (Figure 1B) and 508 down-regulated (Figure 1C) (Table 1) (Supplementary Table S2, Sheet 1). After low-N treatment, 368 DEGs were uniquely identified in the XYT_HNT comparison (region II), of which 169 were up-regulated (Figure 1B) and 199 down-regulated (Figure 1C) (Table 2) (Supplementary Table S2, Sheet 2). Region III shows DEGs that were specifically expressed in HN138 under low-N treatment, among which 198 were up-regulated and 61 were down-regulated (Supplementary Table S2, Sheet 3). Meanwhile, Supplementary Table S2, Sheet 4 lists some of the 59 DEGs shared by XYT_XYC and HNT_HNC, i.e., overlapping/shared DEGs between the two hybrids in response to low-N stress (region IV), among which 41 were up-regulated and 18 were down-regulated (Figure 1B,C). Meanwhile, the low-N-tolerant genotype XY335 had a significantly higher number of DEGs responding to low-N stress (924) than low-N-sensitive genotype HN138 (448), which may suggest a more responsive transcriptome in XY335 than in HN138.

### 3.2. Proteomic Analysis of the Two Maize Genotypes’ Response to Low-N Treatment

**Figure 2:**
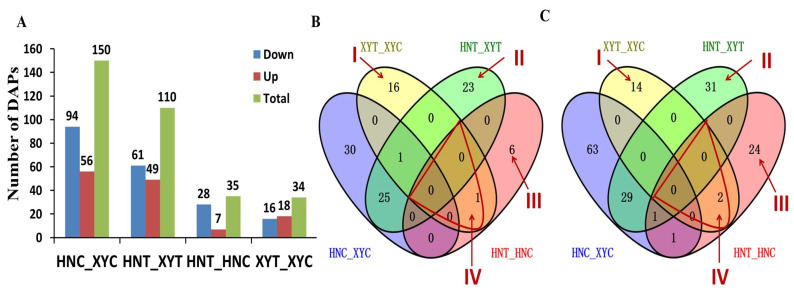
Analysis of differentially abundant proteins (DAPs) identified in four experimental comparisons. (**A**) Number of DAPs identified in each experimental comparison group. Up-regulation-means an increase in the abundance of a DAP. Down-regulation-refers to a DAP with reduced differential abundance; red: up-regulated and blue: down-regulated. (**B**) Venn diagram analysis of up-regulated DAPs. (**C**) Venn diagram analysis of down-regulated DAPs. The overlapping areas of the Venn indicate that there is a DAP overlap between the corresponding groups. Region I shows DAPs specifically expressed in XYT_XYC in response to low-N stress; region II shows DAPs uniquely expressed in the XYT_HNT comparison in response to low-N stress; region III contains DAPs uniquely expressed in HNT_HNC under low-N stress; region IV shows DAPs overlapping between two hybrid lines under low-N stress.

We used the TMT comparative proteomics analysis method to study the changes in XY335 and HN887 leaf protein profiles under low-N conditions. Under the conditions of normal-N (NN) and low-N (LN) stress, we conducted a comparative analysis of the proteomes in the low-N-tolerant hybrid XY335 and low-N-sensitive hybrid HN138. Four experimental comparisons were formed through pairwise comparison (Figure 2A), and a total of 329 DAPs were obtained. The analysis identified 150 DAPs between HN138 and XY335 comparison under control (HNC_XYC), of which 56 were up-regulated and 94 were down-regulated. After low-N treatment, comparison between HN138 and XY335 (XYT_HNT) yielded 110 DAPs which showed differential expression, of which 49 were up-regulated and 61 down-regulated. Comparison between low-N-tolerant hybrid XY335 under low-N treatment and control (XYT_XYC) identified 34 proteins with differential abundances, of which 18 DAPs were up-regulated and 16 were down-regulated. At the same time, 35 DAPs were obtained by comparing the sensitive hybrid HN138 under low-N treatment and control (HNT_HNC), among which seven were up-regulated and twenty-eight were down-regulated. Taken together, the results showed that under low-N treatment, the two genotypes had similar numbers of genotype-specific DAPs responding to low-N stress (XYT_XYC compared with HNT_HNC). However, low-N-tolerant cultivar XY335 had a higher number of up-regulated than down-regulated proteins whereas low-N-sensitive cultivar HN138 had a significantly lower number of up-regulated proteins and more down-regulated proteins under low-N stress (Figure 2B,C). This significant proteomic response difference could explain the major susceptibility differences to low-N stress between the two cultivars. 

The Venn diagrams (Figure 2B,C) show a comparative analysis of the up-regulated and down-regulated DAPs displayed in Figure 2A. We have designated the important DAPs, marked as regions I–IV in Figure 2B,C, as the most relevant to low-N stress in maize. The analysis showed that 30 DAPs were specifically expressed in XY335 under low-N stress (region I), with 16 being up-regulated (Figure 2B) and 14 down-regulated (Figure 2C) (Table 3 and Supplementary Table S3, Sheet 1). Additionally, 54 DAPs were uniquely identified in the XYT_HNT comparison (region II), among which 23 were up-regulated and 31 down-regulated (Figure 2B,C) (Supplementary Table S3, Sheet 2). At the same time, region III comprises 30 DAPs specifically expressed by low-N-sensitive genotype HN138 under low-N treatment, of which six were up-regulated and twenty-four down-regulated (Table 4 and Supplementary Table S3, Sheet 3). Meanwhile, Table 5 lists the three DAPs shared between XYT_XYC and HNT_HNC, that is, DAPs overlapping between the two genotypes under low-N stress (region IV). One of these DAPs (*Zm00001d038929_P001*) was up-regulated and two (*Zm00001d043166_P001* and *Zm00001d048630_P001*) were down-regulated (Figure 2B,C).

### 3.3. GO annotation of Specifically Expressed Genes

**Figure 3:**
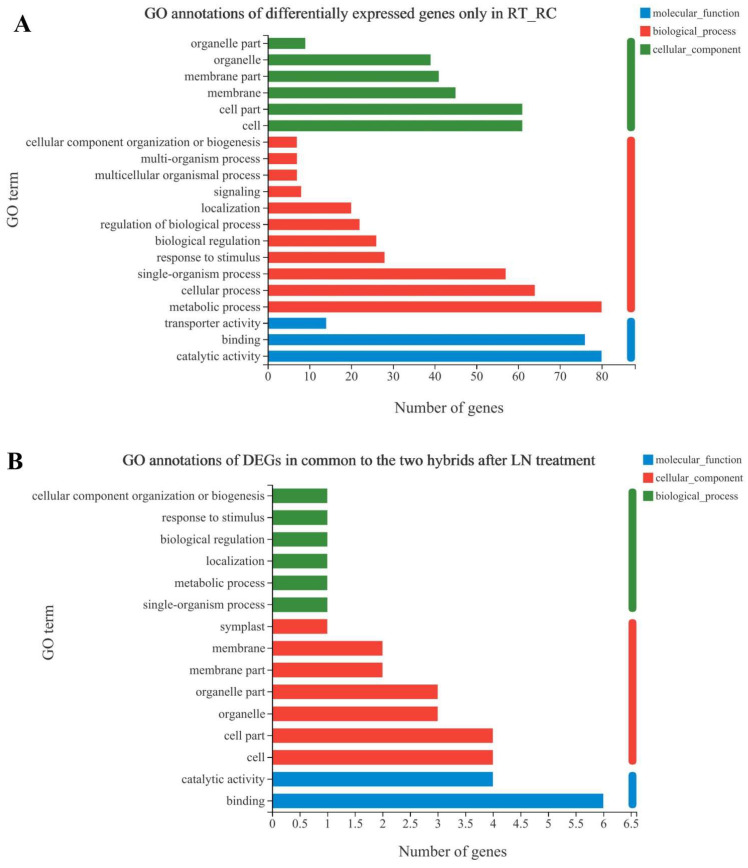
GO annotation of specifically expressed genes, in the low-N-tolerant hybrid XY335 under low-N treatment (**A**); and, commonly shared between the two hybrids under low-N treatment (**B**). The vertical axis in the figure represents the secondary GO classification terms, the upper horizontal axis represents the percentage of genes or transcripts included in the secondary classification, and the lower horizontal axis represents the number of genes/transcripts in the secondary classification in the alignment; the three colours represent the three categories.

We further analyzed the functional roles of the DEGs in the specific expression region I (of the gene set XYT_XYC) using the GO database. The top 20 GO items with the most significant DEGs were classified according to their functional distribution characteristics, and the results are shown in Figure 3. The GO terms mostly annotated in the biological process (BP) category were ‘metabolic process’ (GO:0008152), ‘cellular process’ (GO:0009987), and ‘single-organism process’ (GO:0044699) (Figure 3A). ‘Catalytic activity’ (GO:0003824) and ‘binding’ (GO:0005488) were the most annotated in the molecular function (MF) category. Meanwhile, DEGs localized in ‘cell’ (GO:0005623), ‘cell part’ (GO:0044464), and ‘membrane’ (GO:0016020) accounted for a relatively high proportion.

Additionally, we analyzed the eight genes located in Region IV (Figure 3B), and we found that most BP terms were related to biological regulation and response to stimuli, whereas ‘binding’ (GO:0005488) and’ catalytic activity’ (GO:0003824) were most annotated under the MF category. ‘Cell’ (GO:0005623) and ‘cell part’ (GO:0044464) had a high proportion in the ‘cell component’ (CC) category (Figure 3B).

### 3.4. KEGG Functional Enrichment Analysis of Specifically Expressed Genes

**Figure 4:**
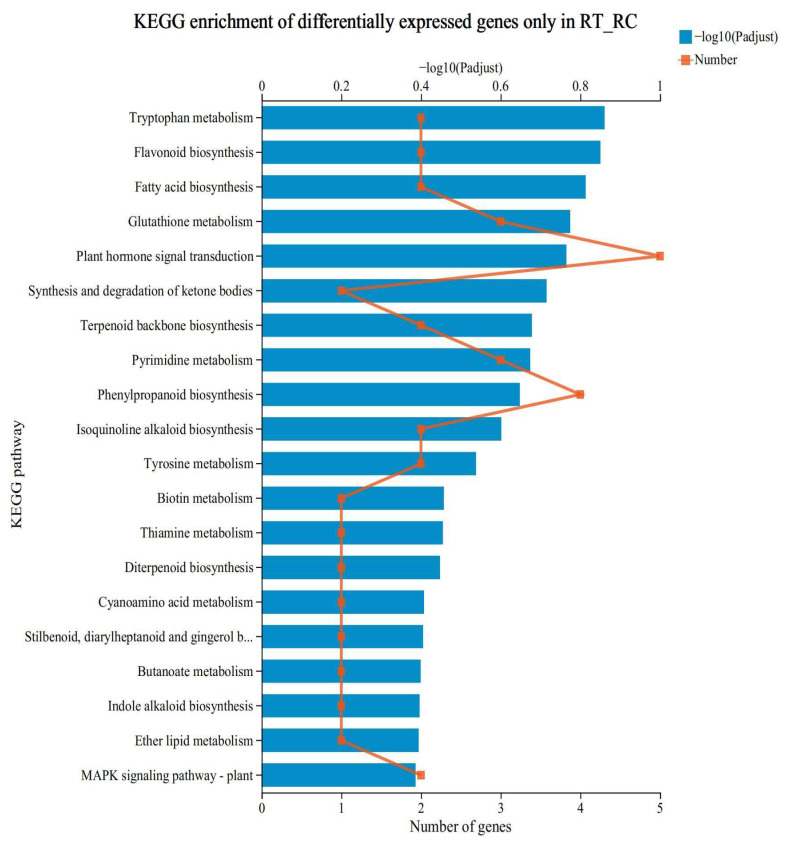
KEGG functional enrichment analysis of DEGs. The ordinate represents the enrichment results of the first 20 KEGG pathways and the lower abscissa represents the number of DEGs in the pathway compared, corresponding to different points on the polyline. The upper abscissa represents the significance level of enrichment, corresponding to the height of the column; the smaller the FDR is and the larger the −log10(*p* adjust) value is, the more significantly enriched the KEGG pathway is.

Figure 4 shows the metabolic pathways that were enriched by the genes specifically expressed in the low-N-tolerant variety after low-N treatment (XYT_XYC). This gene set has special biological significance and may represent the reason why the XY335 variety is more tolerant to low N at the transcriptional level. KEGG enrichment analysis was performed on this group, and the results showed that the top five most significantly enriched pathways were tryptophan metabolism (map00380), flavonoid biosynthesis (map00941), fatty acid biosynthesis (map00061), glutathione metabolism (map00480), and plant hormone signal transduction (map04075).

### 3.5. Transcripts and Proteins Association Analysis

#### 3.5.1. Quantitative Relationship between Transcriptome and Proteome

First, the transcriptome sequencing results of XY335 and HN138 before and after low-N stress were used for CDS (coding sequence) prediction to obtain the protein sequences of these genes. Then, the obtained protein sequence was used as a search database, and the transcriptome and proteome results were searched and aligned with the unified reference genome database (maize B73 reference genome). When a protein was identified and there was expression information at the transcriptome level, it was considered to have a relationship.

After search and alignment, under the screening condition that the abundance of both DAPs and DEGs was ≥1, 3083 genes in the XYT_XYC comparison group were identified at both protein and mRNA levels, and four DAPs were associated with DEGs. The HNT_HNC comparison had 3330 genes that were identified at both protein and mRNA levels, with no associated DAPs and DEGs. 3197 genes were identified at both protein and mRNA levels in the XYT_HNT comparison group, and 10 DAPs were associated with DEGs. 3287 genes were identified at both protein and mRNA levels in the HNC_XYC comparison group, and 23 DAPs were associated with DEGs.

#### 3.5.2. Bioinformatics Analysis of DAPs/DEGs with the Same Expression Trend

GO function significant enrichment analysis was performed on the associated DAPs/DEGs with the same protein and mRNA expression trends to determine the main functions of the DEGs. The results showed that the BP functions of the four DAPs/DEGs derived from the XYT_XYC comparison group were proteolysis and the oxidation-reduction process, and their molecular functions mainly included oxidoreductase activity, serine-type endopeptidase activity, and serine-type peptidase activity. The nine biological processes associated with DAPs/DEGs in the XYT_HNT comparison group included the nitrogen-compound metabolic process and organic-substance biosynthetic process, biological process, and metabolic process. Molecular functions mainly included catalytic activity, transferase activity, and ATP binding. Pathway enrichment analysis was performed on shared DAPs/DEGs using the KEGG pathway database to identify the major metabolic pathways and signal transduction pathways involved in low-N-stress response. The results showed that a total of three pathways were enriched in nine DAPs/DEGs in the XYT_HNT comparison group, which were inositol phosphate metabolism, purine metabolism, and photosynthesis. XYT_HNT was the comparison group under low-N-stress conditions, so the three pathways enriched in this comparison group may be important pathways involved in the response to low-N stress.

### 3.6. Real-Time Quantitative PCR (qRT-PCR) Analysis

**Figure 5:**
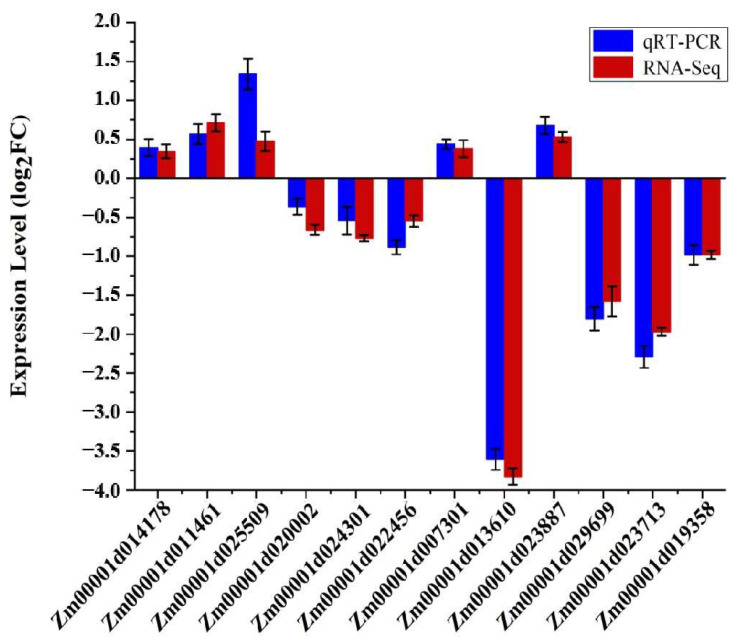
qRT-PCR analysis of different maize low-N-responsive differentially expressed genes (DEGs). All negative expression levels indicate gene down-regulation. *GAPDH* (accession number X07156) was used as an internal reference. The y-axis represents the relative expression level of qRT-PCR (blue; log2-fold change) and log2-fold change of RNA-seq data (red). Error bars represent SE (n = 3).

In order to verify the accuracy of the results of the RNA-seq experiment, we analyzed 12 randomly chosen representative genes that were differentially expressed under low-N stress (listed in Supplementary Table S1) using qRT-PCR analysis. The results showed that the transcription patterns and levels of all the 12 sampled genes were consistent with the RNA-seq results (Figure 5). The correlation coefficient (R^2^, of the fold changes between qRT-PCR and RNA data) was 89% (Supplementary Figure S1). Therefore, the results of qRT-PCR analysis supported the reliability of the transcriptome analysis results.

### 3.7. Function Analysis of ZmGST42 Gene in Low-N-Stress Response

#### 3.7.1. The Effect of Nitrogen Supply Level on Phenotypic Traits of ZmGST42 Mutant

According to our transcriptomic analysis, we observed *ZmGST42* DEG in the XYT_XYC comparison group and as one of the DEGs specifically expressed by the low-N-tolerant hybrid XY335 after low-N stress. Through GO annotation and KEGG enrichment analyses, we found out that the glutathione metabolism pathway was significantly enriched after low-N stress. Therefore, combining our bioinformatics analysis results and information from the published literature, we hypothesized *ZmGST42* as a potential contributor to LN stress tolerance, and we selected it for function verification. 

**Figure 6:**
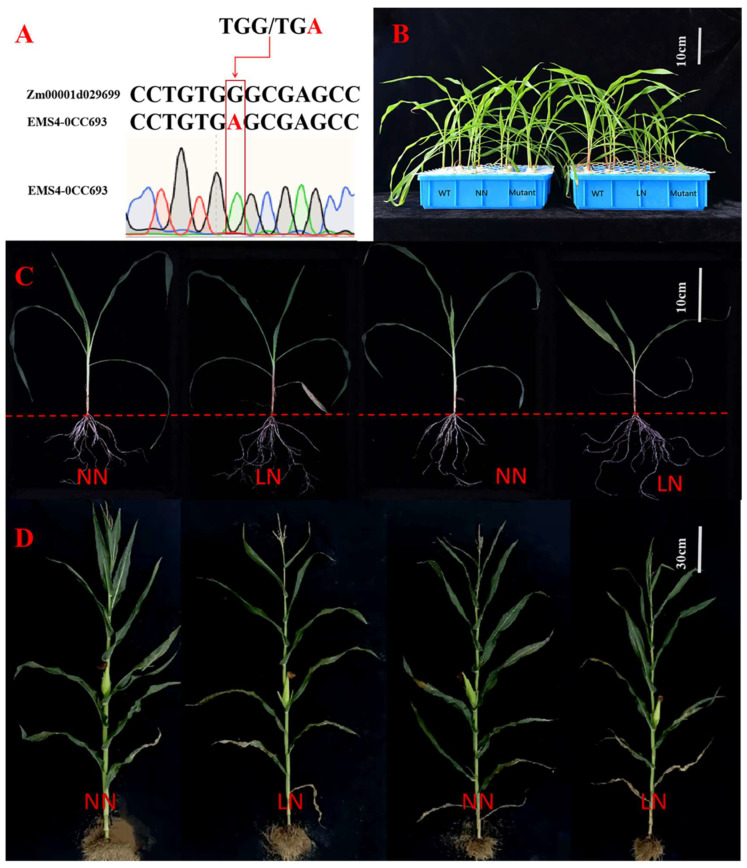
Phenotypic characterization of the WT and mutant plants under low-N stress. The phenotypes presented here are for maize at the seedling and grain filling stages treated with N deficiency. (**A**) The stop codon induced by EMS results in a truncated protein. (**B**) Phenotype control of WT and mutant plants under normal-N (NN) and low-N (LN) treatments at the seedling stage. (**C**) The individual plant phenotype control of WT plant and mutant under normal-N and low-N treatments at the seedling stage. (**D**) The individual plant phenotype control of WT plant and mutant under normal-N and low-N treatments at the grain filling stage. From left to right, (**C**,**D**) are normal-N-treated B73, low-N-treated B73, normal-N-treated mutant, and low-N-treated mutant. WT, wild type; Mutant, mutant; LN, low N; NN, normal N.

Through experiments at the seedling and grain filling stages, we found that LN conditions inhibited the growth of WT maize and the *ZmGST42* mutant. Under LN conditions, WT and mutant plants grew shorter, with small and thin leaves, and the leaves started to turn yellow from the bottoms (Figure 6). The stem thickness in WT and mutant plants was measured by using a vernier caliper, and we found out that the stem thickness under LN conditions was smaller than that under NN conditions (Supplementary Tables S4 and S5). Moreover, under LN conditions, the stem thickness of the mutant was significantly smaller than that of the WT plant under the same treatment conditions (*p*-value < 0.05). Under NN conditions, the difference in stem thickness between WT and mutant plants was not significant (Supplementary Tables S4 and S5). In addition, by measuring the plant height, we observed that under LN and NN conditions, the difference in plant height and green leaf area of the WT plant were not significant. However, the plant height and green leaf area of the mutant under LN and NN conditions were significantly different (Supplementary Tables S4 and S5). In addition, the results of seedling stage experiments showed that the SPAD values of the WT and mutant plants were higher than those under LN treatments. Meanwhile, under LN treatments, the SPAD values of the WT plant were significantly higher than that of the mutant (Supplementary Table S4). By measuring the dry weight and fresh weight of the above-ground and underground parts of the WT plant and mutant under LN and NN conditions, the root-to-shoot ratios of the plants were calculated.

The results at both seedling and grain filling stages showed that the root-to-shoot ratio in the mutant was greater than that in the WT plant at both crop growth stages. However, under the LN treatment, the root-to-shoot ratio of the mutant was significantly greater than that of the WT plant (Supplementary Tables S4 and S5). According to the results of the root scanner at the seedling stage, under NN conditions, the total root length, projected area, root surface area, and root volume of the maize mutant and WT plants were not significantly different. However, under LN conditions, the total root length, projected area, root surface area, and root volume of the mutant and WT plants were significantly different although the average root diameter was not significant. In addition, compared with NN conditions, the growth rate of total root length, projected area, root volume, surface area, and average volume of the mutant under LN conditions were greater than those of the WT plant (Supplementary Table S5).

#### 3.7.2. Effects of Different N Concentrations on the Antioxidant Enzyme Activities and MDA Content of WT and Zmgst42 Mutant Leaves

**Figure 7:**
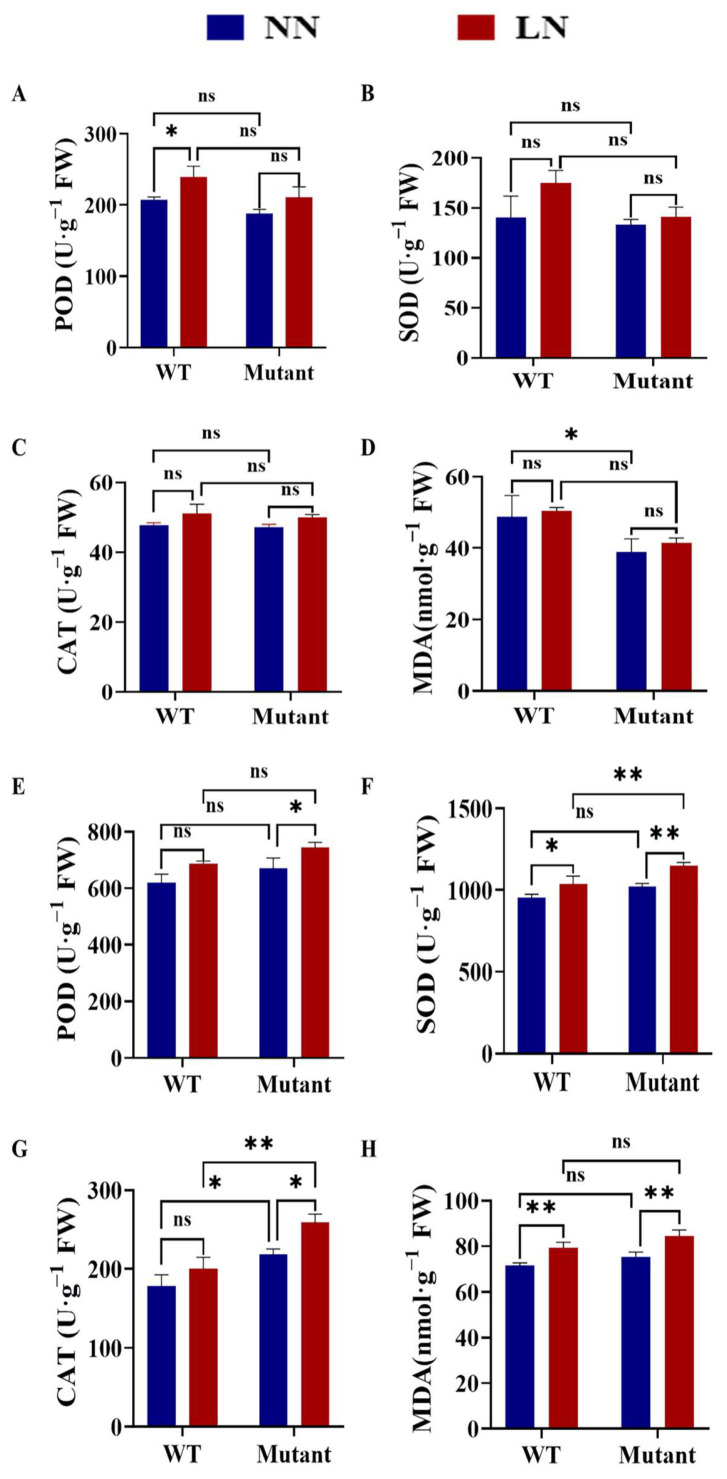
The effect of different N concentrations on the antioxidant enzyme activities and malonaldehyde (MDA) content in wild-type and mutant maize lines at the seedling and grain filling stages. (**A**–**D**) Seedling stage: (**A**) POD, (**B**) SOD, and (**C**) CAT activities and (**D**) MDA content. (**E**–**H**) Grain filling stage: (**E**) POD, (**F**) SOD, and (**G**) CAT activities and (**H**) MDA content. The values shown are means ± SE (n = 6). Significant differences between treatments were analysed using Tukey’s test, * *p* < 0.05, ** *p* < 0.01. Note: WT, wild type; ns, not significant; LN, low N; NN, normal-N conditions.

Under normal-N (control) conditions, at the seedling stage, no significant differences were observed between the WT plant and *gst42* maize mutant in terms of antioxidant enzyme (POD, SOD, and CAT) activities (Figure 7A–C); however, the MDA content values of the WT and mutant plants were significantly different (Figure 1D). Under low-N (LN) treatment, at the seedling stage, the activities of POD, SOD, and CAT in WT and mutant leaves were all increased, and POD, SOD, and CAT were significantly different (Figure 7A–C). The POD, SOD, and CAT activities were significantly higher in the WT plant than in the mutant line (Figure 7A–C). MDA content had a relatively small increase in both WT and mutant plants after LN treatment, but was not significantly different from control conditions (Figure 7D). At the grain filling stage, POD, SOD, and CAT activities were increased in both WT and *gst42* mutant-line plants under LN treatment; however, they were all significantly different in the mutant line, whereas only SOD was significantly different in the WT plant (Figure 7E–G). Meanwhile, MDA content was significantly increased in both WT and mutant plants under LN treatment (Figure 7H), suggesting the effect of low-N stress on lipid peroxidation. However, the increase in MDA content in the WT was smaller than that in the mutant after LN treatment (Figure 7H). Taken together, these results may suggest that low-N stress induced the activation of antioxidant enzymes, possibly to counteract the triggered lipid peroxidation, and this activation was greater in the WT plant than in the mutant. Additionally, the higher lipid peroxidation in the *gst* mutant line than the WT plant may suggest that the *GST42* gene is possibly involved in antioxidant defense under low-N-stress conditions.

#### 3.7.3. Effects of Different Nitrogen Concentrations on the Contents of Relevant Nitrogen Metabolizing Enzymes in ZmGST42 Leaves

The experimental results at both the seedling and grain filling stages showed that after N treatment, compared to NN conditions, the activity of N-metabolizing enzymes in the mutant leaves changed significantly (Supplementary Figure S2). Under the NN and LN conditions, the GST activity in the WT plant was significantly higher than that in the mutant. After LN treatment, the GST activity of the WT plant increased significantly, while the GST activity of mutant decreased slightly. Under LN treatment, the NR activity in both WT and mutant leaves decreased, indicating that leaf NR activity is affected by low-N treatment. Additionally, the WT plant had a smaller decrease in NR enzyme activity than the mutant. Under NN and LN conditions, the GS and glutamate synthetase activities (GOGAT) of WT leaves were higher than those of the mutant leaves. After LN treatment, the NR and GOGAT of WT and mutant plants also decreased, and the NR and GOGAT enzyme activities in the WT plant decreased less than in the mutant line. GSH is the upstream gene of GST. After LN treatment, GSH in the wild type was significantly higher than that in the mutant type. γ-glutamyltranspetidase (γ-GT) is expressed downstream of GST; after LN treatment, WT and mutant γ-GT increased significantly. However, the γ-GT increased more greatly in the mutant than in the WT.

## 4. Discussion

Stress caused by the lack of the N element, especially during the flowering and grain filling stages, has a devastating effect on the final yield of maize [3]. Therefore, understanding how maize responds to low-N stress at the molecular level is of great significance for guiding the genetic improvement of maize low-N tolerance so as to maintain a sustainable high yield under such conditions [39]. In order to fully understand the molecular mechanisms of maize response to low-N stress, and to identify the proteomic and transcriptomic perturbations related to low-N stress at the grain filling stage, this paper compares the low-N susceptibility of two hybrids through integrated transcriptome and proteome analyses. In addition, the function of low-N-stress-related gene *ZmGST42* has been analyzed via experiments on B73 wild-type and mutant plants at the seedling and grain filling stages. Our findings provide a better understanding of the molecular mechanisms of low-N tolerance during the maize grain filling stage.

### 4.1. Low-N-Stress-Tolerance-Related DEGs and DAPs Identified in the Low-N-Tolerant Cultivar XY335 

We performed transcriptomic and proteomic analyses of the maize low-N-tolerant hybrid XY335 to determine its transcriptomic and proteomic responses to low-N treatment. By analyzing these DEGs and DAPs, key candidate genes, proteins, and metabolic pathways that respond to low-N stress were identified.

#### 4.1.1. Genes and Proteins Related to Photosynthesis under Low-Nitrogen Stress

Our proteomic and transcriptomic analyses results showed that photosynthesis was the most significantly enriched metabolic pathway in XY335. The enriched genes included *psbH*, *bZIP-transcription factor 79*, ATP synthase subunit delta chloroplastic, etc., all of which were up-regulated under low-N stress. bZIP transcription factors have been implicated in drought tolerance in maize [40]; however, the exact role of *bZIP-TF79* in low-N-stress response is yet to be ascertained; hence, it will need to be confirmed. The chloroplast-domiciled photosynthesis is one of the main processes affected by abiotic stress [41]. The abiotic stress effects may directly cause the closing of stomata, reduce the diffusion of CO_2_, and also affect the regulation of light. Therefore, the rapid response of the photosynthetic machinery is key for plants to adapt to fluctuating environments [42,43].

#### 4.1.2. RNA Metabolism and Protein Phosphorylation-Related Proteins and Genes in Response to Low-N Stress

Under adverse factors, plants can protect various metabolic reactions in cells by changing their protein structures, thereby maintaining the integrity of plant structures and function [44]. In the current study, several proteins or genes related to RNA metabolism, protein phosphorylation, and homeostasis were up-regulated, including putative DEAD-box ATP-dependent RNA helicase family protein, DEAD-box ATP-dependent RNA helicase 57, leucine-rich repeat protein kinase family protein, etc. (Table 1 and Table 3). DEAD-box RNA helicases constitute the largest subfamily of RNA helicase superfamily 2 (SF2) and are involved in several plant-growth, development, and abiotic-stress-response-related roles [45]. DEAD-box RNA helicases function in many aspects of RNA metabolism [46]. For instance, DEAD-box RNA helicase *AtRH57* may crucially regulate the formation of small ribosomal subunits [47]. In wheat, *TaDEAD-box57-3B* has been suggested to enhance tolerance to drought, salt, and cold stress in transgenic plants by regulating the extent of membrane lipid peroxidation [45]. Possibly, in the current study, DEAD-box RNA helicases, interacting with other proteins/genes, may have been pivotal in regulating membrane lipid peroxidation to enhance low-N-stress tolerance since the MDA content was significantly reduced in the low-N-tolerant cultivar; this will require verification in the future. The enzyme that catalyzes the phosphorylation of proteins is called protein kinase, and it is dependent on intracellular messengers, mediates and amplifies the process of protein phosphorylation, and helps complete the signal transmission process [48,49]. In this study, under low-N stress, leucine-rich repeat protein kinase family protein was up-regulated in its expression, and by catalyzing protein kinases, it helps transmit stress signals and amplify the process of protein phosphorylation [50].

#### 4.1.3. Defense-Related Proteins and Genes under Low-N Stress

In the process of long-term evolution, plants have formed their own defense systems to prevent damages in order to resist environmental stresses that are encountered and not conducive to their own growth and development [51]. Particularly, when maize is subjected to low-N stress, the plant will immediately respond to the stimulus, appropriately increase the metabolic activity in the body, produce defense substances, and enhance the production of various enzymes. Copper transport protein CCH was up-regulated after low-N treatment. Copper transporter is a small molecule transporter responsible for the transmission of copper ions in the cytoplasm, which has the effect of eliminating reactive oxygen species (ROS) during abiotic stress [52,53,54]. Changes in environmental conditions will strongly affect plant productivity, and the accumulation of toxic compounds produced by the interaction between ROS and polyunsaturated fatty acids in the membrane lipids will significantly damage plant cells. Plant aldo-keto reductase (AKR) and other enzymes have been shown to be effective in detoxifying reactive aldehydes produced by lipid peroxidation [55,56]. These findings are consistent with the up-regulated expression of aldo-keto reductase 4 observed in this study in response to low-N treatment. Other key candidate genes/proteins identified in response to low-N stress include PHD finger protein ALFIN-LIKE 6, Ran BP2/NZF zinc finger-like superfamily protein, and zinc finger CCCH domain-containing protein 46 (Table 2), which have been previously implicated in maize abiotic stress response and tolerance [57,58,59]; their exact roles in low-N-stress tolerance will need to be confirmed. 

### 4.2. Differentially Regulated DAPs and DEGs in Low-N-Sensitive Cultivar HN138

In order to conduct a comparative analysis and further determine the main difference between the low-N-tolerant hybrid XY335 and low-N-sensitive hybrid HN138 in response to low-N stress, we also studied the response strategies of HN138. 

#### 4.2.1. Antioxidant-Related Proteins and Genes under Low-Nitrogen Stress

The analysis of low-N-sensitive hybrid HN138-specific DAPs showed that rhicadhesin receptor, GST23, and thioredoxin (TRX) superfamily protein were differentially expressed under low-nitrogen stress. A hair-like protein with the same protein sequence as a rhicadhesin receptor, was found in pea nodules, and the protein has superoxide dismutase activity [60,61]. TRX superfamily protein is a multifunctional protein with redox activity widely existing in plants. It can participate in the electron transfer in photosynthesis, repair of oxidized proteins, and physiological metabolisms such as the elimination of active oxygen [62]. After low-N stress treatment, oxidative damage occurred in maize chloroplasts. Although the increased chloroplast lipoxygenase activity increased lipid peroxidation, plants reduced the oxidative damage by up-regulating the antioxidant defense system in the chloroplast via the TRXs and other antioxidant enzymes.

#### 4.2.2. Proteins and Genes Related to “Stress Response” and “Stimulus Response” under Low-N Conditions

Several proteins and genes with stress response and stimulus response functions under low-N-stress conditions were found in HN138. A number of studies have shown that MCPs play important roles in plant defense response [63,64,65]. MCPs play key roles in developmental regulation and stress-induced programmed cell death, which is essential in many aspects of plant development and in response to various stresses. Non-specific lipid-transfer proteins (nsLTPs), also known as lipid transfer proteins (LTPs), are widely found in plants and are small but basic secreted proteins [66,67,68]. In summary, the nsLTPs and MCPs identified in this experiment produce a stress response under low-N stress and participate in maize’s abiotic stress response.

#### 4.2.3. Other Defense-Related Proteins and Genes in Response to Low-N Stress

Through the analysis of the sensitive hybrid HN138 DAPs and DEGs, we also found 60S ribosomal protein L14-1, which responded to low-N stress. The 60S ribosomal protein is a large subunit of the ribosome. It participates in the translation process in the cytoplasm and has been implicated in plant abiotic and biotic stress responses [69]. In the current study, we observed that low-nitrogen treatment induced the up-regulation of 60S ribosomal protein L14-1, which means low-N stress directly affects HN138’s cell structural integrity and energy metabolism process. 

In this study, in terms of plant phenotype and yield traits, HN138 is less resistant to low-N stress than XY335. However, the up-regulated expression of the above-mentioned types of proteins and genes in leaves induced by low N are all related to the resistance of plants to adversity, which shows that although HN138 has weak resistance to low N, it can still initiate a signal system to resist abiotic stress. 

#### 4.2.4. Key Genotypic Differences in Transcriptomic and Proteomic Responses to Low N Stress

The analysis of genotypic differences in transcriptomic and proteomics responses to low-N stress helps to reveal key candidate low-N-responsive differentially expressed genes and accumulated proteins that can be targeted for functional verification and low-N-tolerance breeding [70,71,72]. Here, our comparative transcriptome analysis showed that higher numbers of DEGs were observed under control than low-N treatment conditions in both genotypes, with XY335 having comparatively higher transcriptome activity [24]. Meanwhile, the low-N-tolerant genotype XY335 had a significantly higher total number of DEGs responding to low-N stress (924) than low-N-sensitive genotype HN138 (448) (Figure 1A), which may suggest a more transcriptome response in XY335 than in HN138. However, this transcriptome response was dominated by more down-regulation of DEGs in XY335 since the two cultivars had almost similar numbers of up-regulated DEGs (Figure 1A). This may suggest that the low-N-tolerant genotype had to suppress the ‘high abundant under sufficient conditions’ or the ‘lavish’ genes in order to minimize lavish growth that may come with more N demand. On the other hand, the minimal down-regulation of lavish genes in the low-N-sensitive cultivar could have resulted in weaker plants that could not better endure N-deficient conditions.

Analysis at the proteome level shows that under low-N treatment, the two genotypes had similar numbers of DAPs responding to low-N stress (XYT_XYC compared to HNT_HNC) (Figure 2A–C). However, low-N-tolerant cultivar XY335 had a higher number of up-regulated than down-regulated proteins whereas low-N-sensitive cultivar HN138 had a significantly lower number of up-regulated proteins and more down-regulated proteins under low-N stress (Figure 2B,C). More importantly, these two genotypes exhibited different sets of up-regulated low-N-responsive DAPs; for instance, leucine-rich repeat proteins were prominent in XY335 but absent in HN138. Instead, antioxidant capacity-related proteins were popular in HN138. This significant proteomic response difference could explain the major susceptibility differences when it came to low-N stress between the two cultivars. On one hand, in addition to the antioxidant system, XY335 enhanced its low-N-stress signal perception and signaling through leucine-rich repeat proteins, which may participate in plant hormone signal transduction and MAPK signaling pathways (Figure 4), and amplified its protein phosphorylation and stress response [73,74]. On the other hand, the antioxidant system activated in HN138 may not have been adequate to alleviate the low-N-stress effects, possibly due to the lack of a robust stress-sensing and signaling mechanism [70]. The functional verification of these differences will improve our understanding of the key mechanisms underlying low-N tolerance in maize. 

### 4.3. Function Analysis of ZmGST42

GST is a multifunctional protease that protects plant cells and protein activity and improves plant resistance; it is a detoxification enzyme that is ubiquitous in various organisms. It can be overexpressed under various biotic or abiotic stresses and enhance the resistance of plants to adversity. It is an important enzyme in the antioxidant defense system [75]. At present, there has been relatively few studies on the response of GST to low-N stress in maize. Therefore, it is of great significance to study the mechanism of *GST* in maize’s response to environmental stresses.

As an antioxidant, GSH plays important roles in plant detoxification and the maintenance of redox buffers. In the process of increasing antioxidant activity, the active oxygen-scavenging system in plants includes two self-protection systems, viz., antioxidant enzyme defense and non-enzymatic antioxidant defense systems [76]. Among them, GSH is the main antioxidant in the non-enzymatic system [77]. GSH is catalyzed by GST to react with toxic substances and convert them into harmless or low-toxicity substances. 

In this experiment, the phenotypic, physiological, and biochemical responses of WT and *ZmGST42* mutant plants to low-N-stress conditions at the seedling and grain filling stages were analyzed. Under LN conditions, the height of the WT and mutant maize plants was stunted, with small and thin leaves; the leaves exhibited yellowing, starting from the bottom and going up. The yellowing of branches and leaves occurred due to the high mobility of N in maize. Nitrogen in the old leaves can be remobilized to the young tissues for reuse after decomposition. Therefore, the leaves turn yellow when there is a lack of N, starting from the lower leaves and gradually moving upward [78]. Nitrogen deficiency also has a certain effect on the chlorophyll content of maize [79,80]. By comparing the mutant and WT plants under LN treatment, we observed that there were more yellow leaf areas, and the plants were shorter, in the WT plant than in the mutant. Under low-N conditions, the SOD, CAT, and POD enzyme activities detected in the WT plant were higher than in the mutant, indicating that the WT plant maintained a higher level of antioxidant enzyme activity to better protect the integrity of its cell membrane [81]. 

Additionally, under LN conditions, the activity of N-metabolizing enzymes in mutant leaves also changed significantly. GST is a detoxification enzyme that is ubiquitous in various organisms. After LN treatment, the GST activity of the WT plant increased significantly while the GST activity of the mutant decreased slightly. Additionally, nitrate reductase (NR) is the first enzyme in the chain of inorganic N assimilation reactions and glutamine synthetase (GS) is a key enzyme in the GS/GOGAT cycle; NR and GS expression have become research hotspots for improving crop N utilization [82]. After LN treatment, the GST activity of the WT plant increased significantly while the GST activity of the mutant decreased slightly. Due to the WT mutation, GST was not expressed, resulting in a decrease in its downstream products, resulting in an increase in the content of γ-GT, both of which promoted the formation of the product. In summary, these results may suggest that *ZmGST42* positively regulates N metabolism under low-N stress. In addition to the physiological and biochemical research on *ZmGST42*, we plan to further study its gene function through transgenic methods and genome editing so as to breed new low-N-tolerant maize varieties. These findings will contribute to the future marker-assisted selection or breeding of highly tolerant materials in low-N environments.

## 5. Conclusions

Here, we have conducted an integrated leaf transcriptome and proteome analysis of two maize hybrids, XY335 and HN138, contrasting in low-N sensitivity at the grain filling stage. We identified different sets of low-N stress-responsive DEGs and DAPs in the two maize cultivars using Venn diagram analysis. At the transcriptome level, the low-N-tolerant genotype XY335 had a significantly higher total number of DEGs than the low-N-sensitive genotype HN138, which may suggest a more transcriptome response in XY335 than in HN138. However, this transcriptome response was dominated by more down-regulation of DEGs in XY335. Key among up-regulated genes/proteins were leucine-rich repeat protein, DEAD-box ATP-dependent RNA helicase family proteins, copper transport protein, and photosynthesis-related proteins. At the proteome level, the two cultivars had similar number (but different sets) of genotype-specific low-N-responsive DAPs. Whilst leucine-rich repeat proteins were prominent in XY335 and absent in HN138, antioxidant capacity-related proteins were popular in HN138; these differences could help explain the low-N susceptibility differences between the two genotypes. Most of the key low-N-responsive genes/proteins identified were involved in the MAPK signaling pathway, regulating membrane lipid peroxidation, and photosynthesis. Our results may suggest that XY335 can better tolerate low-N stress than HN138, possibly through a robust low-N-stress-signal sensing and signaling, amplified protein phosphorylation and stress response, increased photosynthesis efficiency, and down-regulation of ‘lavish’ or redundant proteins to minimize N demand. In addition, we characterized and analyzed the function of low-N-stress response gene *ZmGST42* through physiological and biochemical analyses of the WT plant and its mutant at the seedling and grain filling stages. Under low-N stress, the WT had stronger resistance to low-N stress than its mutant counterpart. The most direct phenotypic differences were in plant height and stem thickness, not the degree of leaf wilting, suggesting that *ZmGST42*’s regulation of low-N tolerance may be related to plant height to a certain extent. Overall, this study has revealed key genes and metabolic pathways that regulate maize low-N-stress tolerance at the grain filling stage and improved our understanding of the molecular mechanisms underpinning low-N tolerance in maize. The candidate genes identified can be targeted for low-nitrogen-tolerance breeding in maize.

## Figures and Tables

**Table 1:** Some major DEGs specific to XY335 in response to low-nitrogen stress.

No.	Gene ID ^1^	Description ^2^	Log_2_FC ^3^	*p* Value ^4^	Expr. ^5^	KEGG Pathway ^6^
1	*Zm00001d024533*	Putative DEAD-box ATP-dependent RNA helicase family protein	8.72	5.39 × 10^−12^	up	Spliceosome
2	*ZemaCp020*	atpA	6.74	2.23 × 10^−5^	up	Oxidative phosphorylation; Photosynthesis
3	*Zm00001d030822*	Cycloartenol synthase	6.52	4.98 × 10^−5^	up	------
4	*Zm00001d022356*	DEAD-box ATP-dependent RNA helicase 57	5.55	5.90 × 10^−4^	up	------
5	*Zm00001d029012*	Leucine-rich repeat protein kinase family protein	1.34	4.25 × 10^−2^	up	------
6	*Zm00001d016691*	Copper transport protein CCH	1.27	4.76 × 10^−2^	up	------
7	*Zm00001d052242*	ATP synthase subunit delta chloroplastic	1.26	4.18 × 10^−2^	up	Photosynthesis; oxidative phosphorylation
8	*Zm00001d005996*	PSI reaction center subunit V	1.24	1.73 × 10^−2^	up	Photosynthesis
9	*ENSRNA049465879*	Eukaryotic small subunit ribosomal RNA	−9.17	1.9 × 10^−2^	Down	------
10	*Zm00001d024751*	Peroxidase 64	−7.83	4.51 × 10^−2^	Down	Phenylpropanoid biosynthesis
11	*Zm00001d020938*	bZIP TF family protein	−5.86	2.89 × 10^−3^	Down	Plant hormone signal transduction
12	*Zm00001d042292*	SAUR-like auxin-responsive protein family	−5.71	1.23 × 10^−3^	Down	Plant hormone signal transduction
13	*Zm00001d029696*	GST U16	−1.74	8.04 × 10^−7^	Down	Glutathione metabolism
14	*Zm00001d004664*	Cytochrome P450 94B3	−1.03	2.85 × 10^−3^	Down	-----
15	*Zm00001d052543*	bZIP TF family protein	−1.42	1.87 × 10^−5^	Down	Plant hormone signal transduction
16	*Zm00001d028816*	PRP 10	−1.88	4.04 × 10^−5^	Down	------
17	*Zm00001d020780*	GST23	−1.13	7.11 × 10^−5^	Down	Glutathione metabolism
18	*Zm00001d040702*	Guaiacol peroxidase1	−1.8	6.90 × 10^−4^	Down	Phenylpropanoid biosynthesis

**Note**: ^1^ Gene ID, unique gene-identifying number in the Maize Genetics and Genomics Database (Maize GDB); ^2^ Gene description; GST, glutathione S transferase; PRP, pathogenesis-related protein; ^3^ Log2FC, log twofold change; ^4^ *p* Value, gene relative expression, up- for up-regulation and down- for down-regulation; ^5^ *p*-value, statistical level (using Student’s *t* test) < 0.05, at which gene differential expression was accepted as significant; ^6^ Pathways, metabolic KEGG pathways.

**Table 2:** Some of the major DEGs uniquely identified in the two maize hybrids after low-N treatment.

No.	Gene ID ^1^	Description ^2^	Log2FC ^3^	*p* Value ^4^	Expr. ^5^	KEGG Pathway ^6^
1	*Zm00001d027580*	Mitochondrial outer membrane protein porin 4	7.83	4.52 × 10^−2^	up	------
2	*Zm00001d049006*	PHD finger protein ALFIN-LIKE 6	6.59	2.36 × 10^−5^	up	------
3	*Zm00001d035336*	Peroxidase superfamily protein	6.42	3.36 × 10^−5^	up	Phenylpropanoid biosynthesis
4	*Zm00001d037976*	FAM91A1-like protein	5.68	2.76 × 10^−2^	up	------
5	*Zm00001d044526*	Ran BP2/NZF zinc finger-like superfamily protein	2.75	1.35 × 10^−6^	Up	------
6	*Zm00001d023299*	Zinc finger CCCH domain-containing protein 46	2.49	6.80 × 10^−4^	Up	------
7	*Zm00001d011123*	Zinc finger protein	2.08	1.36 × 10^−5^	Up	------
8	*Zm00001d045393*	Ran BP2/NZF zinc finger-like superfamily protein	1.65	1.71 × 10^−4^	Up	------
9	*Zm00001d021732*	Cellulose synthase-like protein D3	1.51	3.47 × 10^−6^	Up	------
10	*Zm00001d038252*	potassium channel2	1.1	1.57 × 10^−5^	Up	------
11	*Zm00001d011316*	------	−8.50	3.92 × 10^−4^	Down	------
12	*Zm00001d038619*	------	−5.85	2.15 × 10^−2^	Down	------
13	*Zm00001d052131*	UPF0614 C14orf102-like protein	−5.71	8.04 × 10^−4^	Down	------
14	*Zm00001d002144*	Protein EPIDERMAL PATTERNING FACTOR 2	−5.22	3.670 × 10^−3^	Down	MAPK signaling pathway—plant

For a full description of the column items, please refer to the Table 1 caption above.

**Table 3:** Key DAPs specifically accumulated in low-N-tolerant hybrid XY335 under low-N stress.

No.	Gene ID ^1^	Description ^2^	Log_2_FC ^3^	*p* Value ^4^	Expr. ^5^	KEGG Pathway ^6^
1	*Zm00001d029012*	Leucine-rich repeat protein kinase family protein	0.42	4.25 × 10^−2^	up	------
2	*Zm00001d013034*	40S ribosomal protein S2-1	0.38	1.90 × 10^−2^	up	Ribosome
3	*Zm00001d016691*	Copper transport protein CCH	0.35	4.76 × 10^−2^	up	------
4	*Zm00001d052242*	ATP synthase subunit delta chloroplastic	0.34	4.18 × 10^−2^	up	Photosynthesis; Oxidative phosphorylation
5	*Zm00001d035854*	Alpha-dioxygenase 1	0.33	4.14 × 10^−2^	up	alpha-Linolenic acid metabolism
6	*Zm00001d005996*	Photosystem I reaction center subunit V	0.31	1.73 × 10^−2^	up	Photosynthesis
7	*ZemaCp051*	psbH	0.28	1.64 × 10^−2^	up	Photosynthesis
8	*Zm00001d042308*	60S ribosomal protein L13	0.27	1.26 × 10^−2^	up	Ribosome
9	*Zm00001d042555*	Zinc-binding dehydrogenase family protein	0.27	2.11 × 10^−2^	up	------
10	*Zm00001d029194*	cytochrome P450 family 81 subfamily D polypeptide 8	0.26	3.20 × 10^−5^	up	------
11	*Zm00001d003311*	Probable 2-oxoglutarate-dependent dioxygenase AOP1	−1.03	4.42 × 10^−2^	down	------
12	*Zm00001d050457*	Protein DETOXIFICATION 27	−0.35	2.47 × 10^−2^	down	------
13	*Zm00001d022060*	NAD(P)-binding Rossmann-fold superfamily protein	−0.50	7.03 × 10^−3^	down	------
14	*Zm00001d031666*	Probable aldo-keto reductase 4	−0.30	1.33 × 10^−2^	down	------
15	*Zm00001d039732*	Staphylococcal-like nuclease CAN1	−0.32	4.32 × 10^−3^	down	------

**Note**: ^1^ Accession, unique protein identifying number in the UniProt database; ^2^ Biological function description; ^3^ log2FC represents the logarithmic value of protein intensity (log 2); ^4^ *p* value, statistical level (using Student’s *t*-test) < 0.05, at which protein differential expression was accepted as significant; ^5^ Expr., Up, up-regulated; Down, down-regulated. ^6^ Pathways, metabolic KEGG pathways.

**Table 4:** Major DAPs accumulated specifically in low-N-sensitive hybrid HN138 in response to low-N stress.

No.	Gene ID ^1^	Description ^2^	Log2FC ^3^	*p* Value ^4^	Expr. ^5^	KEGG Pathway ^6^
1	*Zm00001d033061*	Protein DETOXIFICATION 40	0.27	9.34 × 10^−3^	up	------
2	*Zm00001d025857*	60S ribosomal protein L14-1	0.30	3.65 × 10^−3^	up	Ribosome
3	*Zm00001d004466*	Protein transport protein Sec24-like	0.43	1.57 × 10^−2^	up	PPER
4	*Zm00001d046569*	Protein kinase superfamily protein with Phox/Bem1p domain	0.43	4.43 × 10^−2^	up	------
5	*Zm00001d018954*	Rhicadhesin receptor	0.37	7.77 × 10^−3^	up	------
6	*Zm00001d002000*	lipoxygenase6	0.28	3.35 × 10^−2^	up	Linoleic acid metabolism
7	*Zm00001d004089*	PRP1	−0.68	1.59 × 10^−2^	down	------
8	*Zm00001d038911*	Non-specific lipid-transfer protein 2	−0.60	3.92 × 10^−2^	down	------
9	*Zm00001d042143*	glucan endo-13-beta-glucosidase homolog1	−0.50	9.34 × 10^−3^	down	------
10	*Zm00001d020780*	glutathione transferase23	−0.45	4.76 × 10^−2^	down	Glutathione metabolism
11	*Zm00001d021119*	Beta-glucosidase 11	0.82	4.2 × 10^−2^	down	------
12	*Zm00001d004089*	Pathogenesis-related protein 1	0.63	1.59 × 10^−2^	down	------

**Note**: ^2^ Description: GST, glutathione S transferase; PRP1, pathogenesis-related protein 1; ^6^ KEGG Pathway: PPER, protein processing in endoplasmic reticulum. For a full description of the other column items, please refer to the Table 3 caption above.

**Table 5:** Overlapping low-N-responsive DAPs identified between HN335 and HN138 under low-N stress.

No.	Gene ID ^1^	Description ^2^	Log2FC ^3^	*p* Value ^4^	Expr. ^5^	KEGG Pathway ^6^
1	*Zm00001d038929*	ATP synthase 2	1.36	0.0041	up	Oxidative phosphorylation
2	*Zm00001d043166*	UDP-glycosyltransferase 87A1	0.81	0.0319	down	------
3	*Zm00001d048630*	----	0.24	0.0190	down	------

For a full description of the column items, please refer to the Table 3 caption above.

## Data Availability

All of the data is contained within the article and the Supplementary Materials.

## Supplementary Materials

The following supporting information can be downloaded at: https://www.mdpi.com/article/10.3390/antiox12101831/s1. The mass spectrometry proteomics data have been deposited into the ProteomeXchange Consortium via the PRIDE partner repository with the dataset identifier PXD028027 and the transcriptomic data have been deposited into the NCBI Sequence Read Archive (SRA) with the dataset identifier SRP341978.
