# Supplementary material for: Integrated Transcriptomic and Proteomic Analyses of Low-Nitrogen-Stress Tolerance and Function Analysis of ZmGST42 Gene in Maize
Source: Antioxidants (Basel). 2023 Oct 5;12(10):1831. doi: 10.3390/antiox12101831 (PMC10603844; doi:10.3390/antiox12101831)
Supplement: Supplementary file 1 [file antioxidants-12-01831-s001.zip › Supplementary Figures 1 and 2.pdf]

Supplementary Material

Supplementary Figures 1 and 2

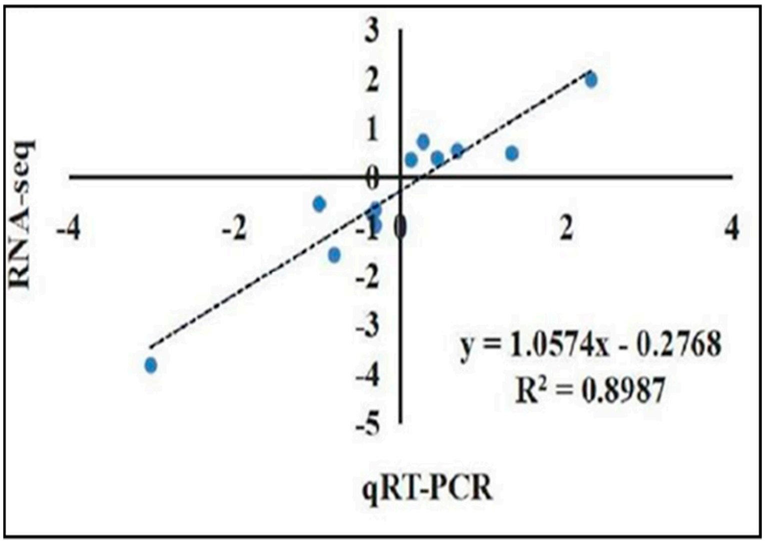

Supplementary Figure 1. Validation of RNA-seq expression data through qRT-PCR analysis. The plots demonstrate the expression ratio in Log scale with base of two. The x-axis is the qRT-PCR logarithmic scale; the y-axis is the RNA-seq Logarithmic scale.

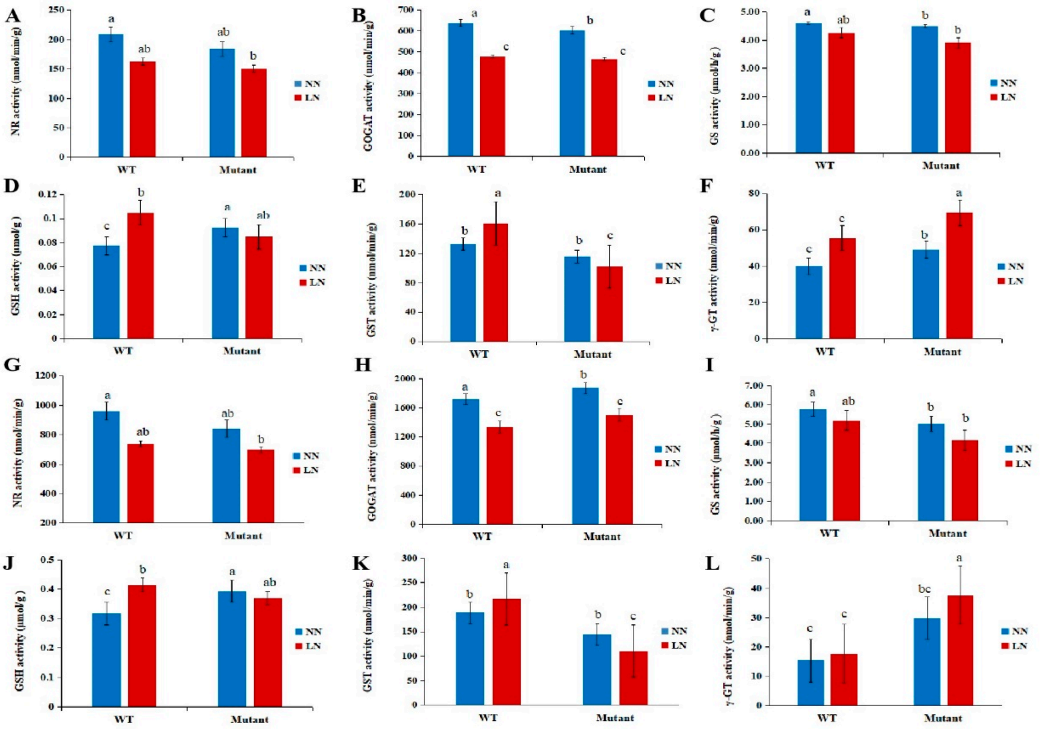

Supplementary Figure 2. The effect of different N concentrations on the content of related N metabolizing enzymes in WT and mutant maize. (A) NR activity; (B) GST activity; (C) GS activity; (D) GSH activity; (E) GST activity; (F) γ-GT activity; (G) NR activity; (H) GST activity; (I) GS activity; (J) GSH activity; (K) GST activity; (L) γ-GT activity. A-F is the seedling stage experiment, and G-L is the grain filling stage experiment. These values are mean ± SE (n = 6). Different letters indicate significant differences between treatments after Tukey's test (P <0.05). WT, wild type; Mutant, mutant; LN, low N; NN, normal N conditions.
