# Supplementary material for: Integrated Transcriptomic and Proteomic Analyses of Low-Nitrogen-Stress Tolerance and Function Analysis of ZmGST42 Gene in Maize
Source: Antioxidants (Basel). 2023 Oct 5;12(10):1831. doi: 10.3390/antiox12101831 (PMC10603844; doi:10.3390/antiox12101831)
Supplement: Supplementary file 1 [file antioxidants-12-01831-s001.zip › Supplementary Tables 1 , 4, and 5.pdf]

Supplementary Material

Supplementary Tables 1, 4, and 5

Supplementary Table 1. Genes-specific primers used for qRT-PCR

No.	Gene name	Forward primer	Reverse primer	Product length	TM
1	Zm00001d014178	CGATGTTACTCAAGTGGTACCT	TCAACCGATAGTATCACTTCGG	168	58.13 58.02
2	Zm00001d011461	CTAAAAGTACGTGCTTCCCATG	CGTACTGTACGTCACTTTTTGG	82	57.98 58.2
3	Zm00001d007301	CGAGGAAGTTGTGATTGCTAAG	CCTTGCTCTTGTTGATGAAGTC	170	57.98 58.16
4	Zm00001d025509	ACAAGCACTACAACTTCGAGTA	TAGGTTCAGAGTACAAGAGCAC	89	58.01 57.8
5	Zm00001d013610	CAACAAGTGCGCTTACACC	GACGAGGGAGATGTCGAAC	283	58.1 57.94
6	Zm00001d023713	CCACACGATCAAAAACAAAACC	GATGAAGCTGGAGTTCATGATG	123	57.8 57.76
7	Zm00001d019358	TTTTACCATTCATTAGCTGCCG	CAAACACGACAAATGCATGATG	123	57.95 58.01
8	Zm00001d023887	CAACATAACAGTCGACGAAACA	GGCAACACGAACAAATTAACAC	174	57.87 57.88
9	Zm00001d020002	GTAACAACAGCTTTTGGTACGG	TGTCAAATACTCAGGGTAGTCG	80	58.69 57.87
10	Zm00001d024301	GACGAGGATGTTCTACGAGATC	TATCCTCAGCGTCTTCGACTTC	91	58.44 60.23
11	Zm00001d022456	GGAAGCTCAACTCGTCCTAATA	AGTAGTTTCCAGATGTATGCGT	100	57.94 57.85
12	Zm00001d029699	CAATCAAAGCAAGCAAAAGAGG	GCCGGGGAGTCTATCTTCTTAT	92	57.59 59.43

Notes:

1) Primers were designed using Primer Premier 5 Designer software.

Supplementary Table 4. The effect of nitrogen supply level on various morphological traits in different maize material at the seedling stage

**Nitrogen application rate**	**LN**		**NN**	
**Variety**	**WT**	**Mutant**	**WT**	**Mutant**
Plant height (cm)	48.23a	36.63b	50.15a	46.67a
Stem thickness (mm)	3.79b	2.62c	4.61a	4.50a
Leaf area	28.17b	15.53c	37.42a	38.21a
SPAD	20.60b	11.29c	34.25a	34.15a
Fresh weight above ground (g)	3.88b	2.19c	5.36a	4.40b
Dry weight of above ground (g)	0.92a	0.73b	1.01a	0.94a
Fresh weight underground (g)	1.73a	1.74a	1.68a	1.95a
Underground dry weight (g)	0.54a	0.60a	0.38b	0.35b
Fresh weight root shoot ratio (%)	45b	80a	31c	44b
Dry weight root to shoot ratio (%)	58b	81a	38c	37c
Root length (cm)	24.68b	32.74a	22.24c	22.60bc
Total root length (cm)	259.97ab	259.97a	238.38b	231.41b
shadow area (cm^2)	161.25a	127.12b	125.15b	122.90b
Root surface area (cm^2)	462.78a	393.32b	390.83b	387.30b
The average diameter (mm)	5.35b	5.03b	6.61a	6.04ab
Root volume (cm^3)	78.57a	56.19b	51.55b	51.34b

Note: Significance level is $P<0.05$.

Supplementary Table 5. The effect of nitrogen supply level on various morphological traits in different maize materials at the grain-filling stage

**Nitrogen application rate**	**LN**		**NN**	
**Variety**	**WT**	**Mutant**	**WT**	**Mutant**
Plant height (cm)	225.66b	184.67c	229a	227.67ab
Stem thickness (mm)	26.43a	20.6c	26.86a	24.29b
Leaf area	507.71b	433.76c	534.11a	529.33a
Fresh weight above ground (g)	739.56a	496.3c	747.2a	682.06b
Dry weight of above ground (g)	381.44b	324.34c	453.67a	448.34a
Fresh weight underground (g)	86.3a	81.42b	84.59a	78.81c
Underground dry weight (g)	25.33a	25.42a	23.59a	22.85b
Fresh weight root shoot ratio (%)	11.67a	16.41b	11.32a	11.56a
Dry weight root to shoot ratio (%)	6.64b	7.84a	5.21c	5.10c

Note: Significance level is $P < 0.05$.
